# Transmission Dynamics and Parameters for Pertussis during School-Based Outbreak, South Korea, 2024

**DOI:** 10.3201/eid3107.241643

**Published:** 2025-07

**Authors:** U. Jin Cho, Seonghui Cho, Hyeokjin Lee, Seul-Ki Kang, Bryan Inho Kim, Yukyung Nam, Chiara Achangwa, Jun-Sik Lim, Dong Han Lee, Sukhyun Ryu

**Affiliations:** Gyeongnam Regional Center for Disease Control and Prevention, Busan, South Korea (U.J. Cho, H. Lee, D.H. Lee); The Catholic University of Korea, Seoul, South Korea (S. Cho, Y. Nam, C. Achangwa, S. Ryu); Korea Disease Control and Prevention Agency, Cheongju-si, South Korea (S.-K. Kang, B.I. Kim); Korea University, Seoul (S.-K. Kang, Y. Nam); National University of Singapore, Singapore (J.-S. Lim)

**Keywords:** pertussis, respiratory infections, bacteria, serial interval, superspreading, pertussis vaccine, effectiveness, epidemic, school, South Korea

## Abstract

We estimated the serial interval and superspreading potential to quantify pertussis transmission dynamics in a 2024 school-based outbreak of a population in South Korea that had received a series of pertussis vaccinations. We analyzed 48 cases of pertussis and reconstructed 36 transmission pairs. We then used maximum-likelihood estimation to assess serial interval and offspring distribution from transmission pair data. We identified that the mean serial interval was 9.5 (SD 1.6) days; 15% (95% CI 8%–23%) of cases seeded 80% of all transmissions in this outbreak. Our findings suggest that pertussis was highly transmissible in vaccinated children during this outbreak. Rapid contact tracing and strict adherence to public health measures are needed to reduce community pertussis transmission.

Pertussis, also known as whooping cough, is a highly contagious respiratory infection caused by *Bordetella pertussis*, a gram-negative bacterium (*1*,*2*). *B. pertussis*, which is found only in humans, accumulates in the respiratory tract and is mainly transmitted through respiratory droplets (*1*,*3*). The distinguishing clinical characteristic of pertussis is a cough that gradually develops into a severe hacking cough, which can persist for several weeks (*1*).

After immunization programs began in the 1940s, the incidence of pertussis was reduced globally (*2*–*4*). However, since the 1980s, a resurgence in pertussis cases has been observed in countries (*2*,*4*). In 2024, the United States and Europe saw an increase in the number of reported cases (*5*,*6*). Although East Asia, including Taiwan, Singapore, and Japan, has not seen an increased number of the cases in 2024 compared to those of 2020–2023 (*7*), in South Korea, pertussis cases have risen sharply, particularly among school-age children (*7*,*8*). Pertussis vaccines are available, including the DTaP (diphtheria, tetanus, and acellular pertussis) vaccine for children and the Tdap (tetanus, diphtheria, and acellular pertussis) vaccine for adolescents and adults. Those vaccines are effective in preventing severe pertussis, but recipients may experience waning immunity acquired from the vaccination over time (*9*,*10*). In South Korea, pertussis has been managed as a notified disease since 1954; a national immunization program provides DTap vaccine at 2, 4, and 6 months of age; between 15 and 18 months of age; and between 4 and 6 years of age. Furthermore, a booster dose of Tdap is recommended for adolescents 11–12 of age in South Korea.

Identifying epidemiologic characteristics provides valuable information for public health practitioners and the public. However, pertussis’s epidemiology and transmission dynamics, including its superspreading potential in a vaccinated population, remain unclear. In March 2024, a pertussis outbreak occurred in a boarding school in Busan, a city in southeastern South Korea with a population of 3.2 million (*11*). The Korea Disease Control and Prevention Agency (KDCA) implemented active case finding under the test-trace-isolate strategy to control the outbreak. In this study, we analyzed the case data associated with this pertussis outbreak and explored the epidemiologic characteristics and transmission dynamics, including the superspreading potential of pertussis from an outbreak that occurred in the school setting. The Institutional Review Board of KDCA waived ethics approval for this study (Institutional Review Board no. KDCA-2024-09-06).

## Methods

### Epidemiologic Investigation

On April 17, 2024, the local education and public health authority in Busan, including the Gyeongnam Regional Center for Disease Control and Prevention, initiated an epidemiologic investigation to identify possible pertussis cases in the school, screening persons who had been in any contact with the case-patient or had any respiratory illness. The public health authority staff interviewed those persons to collect information including demographics, symptom onset date, exposure to the case-patient, and DTaP vaccination history. When vaccination history was unavailable through the interview, public health authorities obtained it from the vaccine registry database of the KDCA. We defined a case as illness in a person who had confirmed *B. pertussis* by laboratory PCR in accordance with KDCA guidelines (*12*). All specimens from all suspected carriers were collected with nasopharyngeal swabs and were tested using a KDCA-approved commercial kit that targets gene coding for an insertion element (IS481) and the pertussis toxin promoter (*12*). We defined controls as persons at the school who tested negative by PCR during the outbreak. We defined the first day of illness as the onset of coughing or cough-preceding cold symptoms. We traced subsequent transmission back to the case-patient on the basis of the epidemiologic investigation report. Then, using contact tracing data and line lists, we established a transmission network (*13*). We determined the transmission network by the timing of exposure of infector and the symptom onset of infectee. When we could not definitively determine transmission pairs based on symptoms and exposure history alone, we investigated shared environments, such as classroom and dormitory interactions, to strengthen the epidemiologic link. Furthermore, we calculated the attack rate as the proportion of persons confirmed as cases during the outbreak out of the total number of persons at risk for infection in the school.

As a response to this outbreak, case-patients were required to stay home until they completed antimicrobial drug treatment. For the person in close contact with the cases, additional pertussis vaccination and preventive drugs (e.g., macrolides) were recommended in accordance with KDCA guidelines. All persons in the school were recommended to wear face masks until June 24, 2024, when the KDCA declared the outbreak over.

### Serial Interval

Serial interval is an essential epidemiologic parameter that describes the time delay between consecutive generations of infected persons. We examined cases with symptom onset dates and reconstructed the transmission pairs by identifying the infector–infectee pair from the established transmission network. We computed the serial interval as the number of days between the symptom onset of the infector and the symptom onset of infectee. Then, we fitted 3 different parametric distributions (log-normal, gamma, and Weibull), which are right-skewed continuous probability distributions, using maximum-likelihood estimation (*14*). We also opted for the best-fitted distributions for serial intervals based on the Akaike information criterion (AIC), in which lower values indicate a better-fitted model. We compared the mean serial intervals between 2 groups of infectors: those vaccinated with DTaP within the previous 5 years and those vaccinated >5 years ago. In addition, we examined the differences in the mean serial intervals between infectors who had received final vaccination doses of 4–5 doses or 6 doses total. We performed comparisons using the Student *t*-test.

### Superspreading Potential

We generated the observed offspring distribution by calculating the number of secondary cases. We fitted the offspring distribution to negative binomial distributions. Then, we presented the parameters, including the effective reproduction number (R_t_), which is the mean number of infected persons resulting from an infector, and overdispersion (*k*), which indicates the individual level of heterogeneity in transmission (*15*). We also compared the *k* between the 2 infectors’ groups (<5 years and >5 years from the vaccination). We examined the statistical significance of the difference using a bootstrap method, resampling with replacement from the fitted negative binomial models to construct 10,000 bootstrap samples (*16*). We then calculated 95% CIs from the 2.5th and 97.5th percentiles of the resulting distribution of differences in *k* values. Furthermore, we estimated the expected proportion of cases responsible for 80% of the total secondary transmission using the estimated R_t_ and *k* obtained from this study (*17*,*18*).

### Waning of Tdap Effectiveness

In South Korea, after the fifth dose of DTaP in children <7 years of age, a booster dose of Tdap with smaller concentrations of pertussis antigens (*19*) is recommended in children 11–12 years of age (*20*,*21*). We assessed the waning of protection against pertussis after the sixth dose of the pertussis vaccine (i.e., Tdap vaccination) by comparing case-patients with controls in this outbreak. To assess the effect of time since vaccination (i.e., the number of years elapsed since the sixth dose of vaccination) on the odds of acquiring pertussis, we used logistic regression models (*9*). We first modeled time since vaccination as an ordinal categorical variable, assuming a structured trend (i.e., monotonic relationship) between the number of years postvaccination and the odds of a positive pertussis PCR test. Furthermore, to assess the validity of this assumption and explore potential nonlinear trends in waning immunity, we employed an unordered categorical model, estimating separate odds ratios for each year following vaccination. By comparing the results from these models, we assessed the appropriateness of assuming a consistent trend in waning immunity over time. Furthermore, in the model, we included covariates to adjust for sex. We performed all statistical analyses in R version 4.4.1 (The R Project for Statistical Computing, https://www.r-project.org).

## Results

### Characteristics of the Study Population

**Figure 1:**
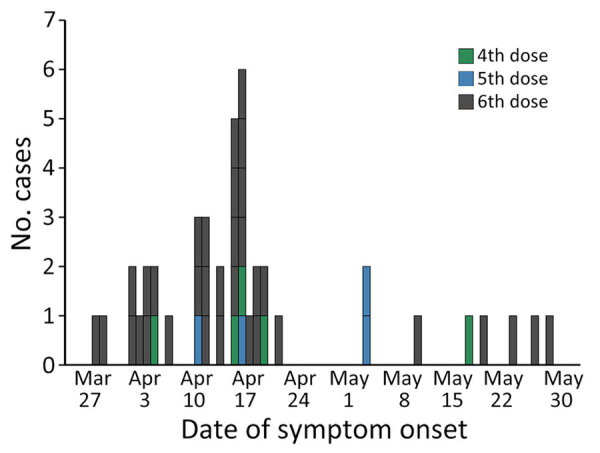
Epidemic curve of daily cases, by symptom onset and number of vaccine doses received, in outbreak of pertussis in a boarding school, Busan, South Korea. Asymptomatic cases (n = 5) were excluded.

This outbreak started on April 2, 2024, and lasted until May 28, 2024. The overall attack rate in the study was 10.1% (48 cases/476 persons). Of the 48 case-patients, 34 (70.8%) were male and 14 (29.2%) female, and the overall median age was 15 years (range 12–18 years) (Table; Figure 1). There were 5 (10.4%) case-patients who had received the fourth dose of the pertussis vaccine, 4 (8.3%) who had received the fifth dose, and 39 (81.3%) who had received the sixth dose. There were 43 (89.6%) symptomatic infections and 5 (10.4%) asymptomatic cases (Appendix Table 1). The median delay between symptom onset and laboratory confirmation was 6 days (interquartile range 3.0–12.3 days).

**Table:** Demographic characteristics of confirmed pertussis cases and controls in a boarding school in Busan, South Korea, 2024

Characteristic	Total	No. cases (%)	No. controls (%)	p value*
Overall	400	48 (12)	352 (88)	
Sex				0.69
M	269	34 (70.83)	235 (66.76)	
F	131	14 (29.17)	117 (33.24)	
Age group, y				0.43
12–15	134	19 (39.58)	115 (32.67)	
16–18	266	29 (60.42)	237 (67.33)	
Most recent vaccination				0.36
1st dose	5	0	5 (1.42)	
3rd dose	1	0	1 (0.28)	
4th dose	31	5 (10.42)	26 (7.39)	
5th dose	39	4 (8.33)	35 (9.94)	
6th dose	321	39 (81.25)	282 (80.11)	
Unavailable†	3	0	3 (0.85)	
Years since last vaccination				0.18
0–4	228	26 (54.17)	202 (57.39)	
5–9	138	20 (41.67)	118 (33.52)	
10–15	31	2 (4.17)	29 (8.24)	
Not available	3	0	3 (0.85)	

*Calculated by χ^2^ test, Mann-Whitney U test, or Student *t*-test, where appropriate. †Vaccine history could not be obtained either from the persons or from the vaccine registry database of the Korea Disease Control and Prevention Agency.

### Serial Interval

**Figure 2:**
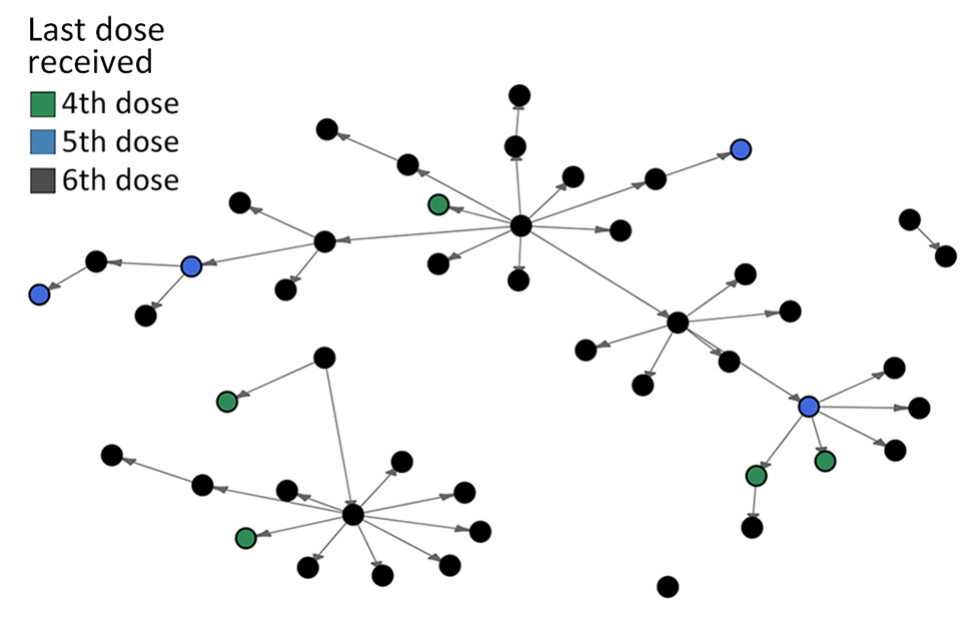
Transmission network of persons who received 4th, 5th, and 6th doses of the DTaP (diphtheria, tetanus, and acellular pertussis) vaccine in outbreak of pertussis in a boarding school, Busan, South Korea. The person with no epidemiologic link was the case-patient identified through laboratory confirmation who had no known contact with another case.

**Figure 3:**
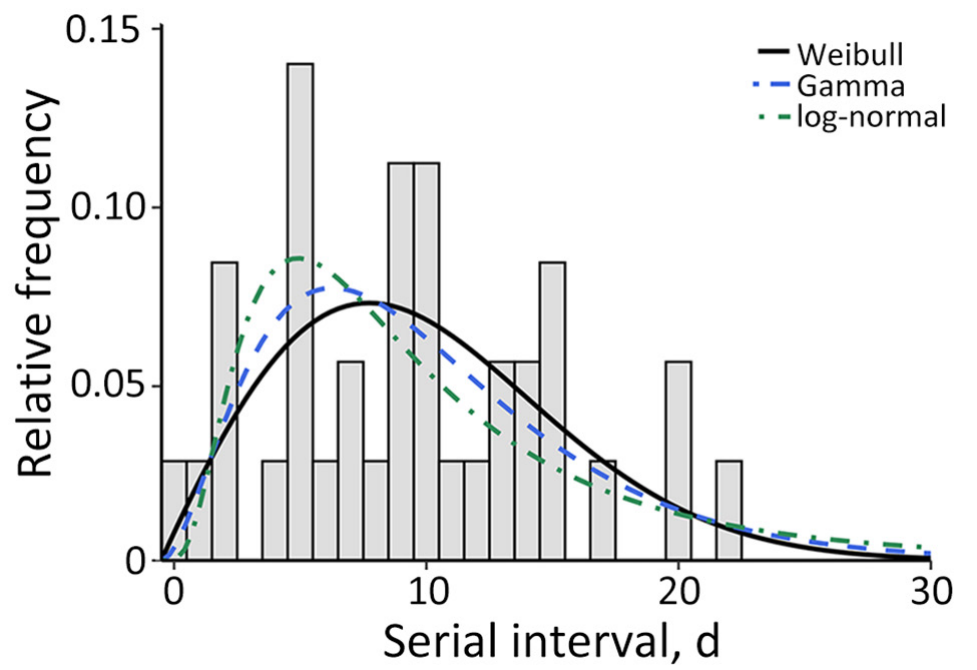
Estimated serial interval distribution associated with pertussis outbreak in a boarding school, Busan, South Korea. We fitted 36 infector–infectee pairs to a Weibull, gamma, and log-normal distribution. Bars indicate frequency of the serial interval; lines indicate the estimated distribution.

We identified 36 transmission pairs with a symptom onset date for both infector and infectee (Figure 2; Appendix Table 1). The Weibull distribution was the best fit for the serial interval distribution, estimated as a mean of 9.5 (95% CI 8.9–10.0) days; SD was 1.6 (95% CI 1.7–2.3) days (Figure 3; Appendix Table 2). We could not identify significant differences in the serial interval between the infector vaccinated within 5 years and those vaccinated >5 years ago, or between infectors with final vaccination doses of 4–5 (p = 0.72) and 6 (p = 0.69) (Appendix Figure).

### Superspreading Potential

**Figure 4:**
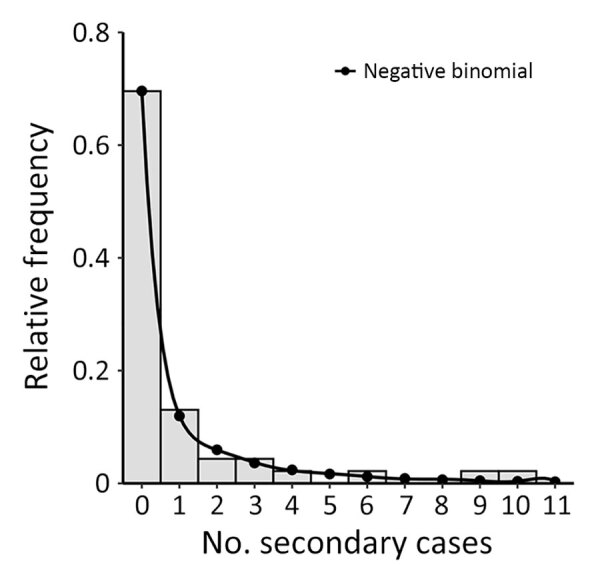
Estimated offspring distribution of cases associated with pertussis outbreak in a boarding school, Busan, South Korea. The offspring distribution is fitted to a negative binomial distribution using effective reproduction number = 0.93 and overdispersion *k* = 0.23.

The overall R_t_, estimated from the observed offspring distribution and negative binomial distribution, was 0.93 (95% CI 0.41–1.85), and *k* was 0.23 (95% CI 0.11–0.53) (Figure 4; Appendix Table 3). We identified *k* of 0.44 (95% CI 0.12–10.27) for persons who had the latest vaccination doses <5 years and 0.38 (95% CI 0.11–6.00) for those who had the latest dose >5 years previously (Appendix Table 4). The difference in *k* between the 2 groups was not statistically significant; bootstrap 95% CI for the difference was −0.11 to 0.24. We estimated that 15.4% (95% CI 8.0%–22.8%) of cases were responsible for 80% of all transmission in this outbreak.

### Waning of Tdap Effectiveness

In the ordered and unordered categorical model for the time since vaccination, the odds ratio (OR) of acquiring pertussis fluctuated across the years. In the ordered model, OR was 0.39–4.72, and in the unordered model, OR was 0.36–3.98. Results for both models had wide CIs and were not statistically significant (Appendix Table 5).

## Discussion

Countries in which a large portion of the population has received a series of pertussis vaccines have reported increased cases since late 2023. In our study, we identified the transmission characteristics of pertussis, including its serial interval and superspreading potential, using contact tracing data in South Korea.

The serial interval is an important factor in determining infectious disease control that is directly correlated with the infection spread rate throughout the community (*22*,*23*). We found that the gamma distribution had a similar mean serial interval with comparable AIC values to the Weibull distribution (Appendix Table 2), which indicated that our serial interval estimate is reliable and that both gamma and Weibull distributions are plausible models for our serial interval data. Previous studies in the United Kingdom, before pertussis vaccine was introduced, demonstrated that the median serial interval was 7 (range 4–56) days (*24*); another study in the Netherlands demonstrated the mean was 23 (95% CI 22–24) days (*25*). Our mean serial interval of 9.5 days is smaller than that determined in previous research (*25*), probably because of differing case definitions; the earlier study accounted for only cases with persistent coughing for >2 weeks. However, because most pertussis cases in South Korea seek care for mild respiratory illness (48%) and atypical cough (i.e., 4% had whooping cough, and 10% had night cough and paroxysmal cough) symptoms (*8*), we defined illness in any person with respiratory illness who had contact with a person with a positive PCR test result as a case. We also found that there was no significant difference in serial intervals according to the postvaccination time of infectors (i.e., 5 years before and after vaccination). Additional study is needed to determine whether the serial interval varies across settings, such as in households.

Regarding superspreading potential, a previous modeling study demonstrated that irregular pertussis epidemics may arise because of stochasticity and nonlinearity in transmission (*26*). Our *k* suggests that pertussis transmission is highly overdispersed; hence, this pertussis outbreak had substantial potential for superspreading events. The *k* in our study was comparable to other outbreaks of respiratory viruses in South Korea: 0.26 for Middle East respiratory syndrome coronavirus (*27*) and 0.10 for the SARS-CoV-2 Omicron variant (*28*). Therefore, enforcing monitoring and physical distancing in high-risk settings and promptly identifying and isolating cases should be the main strategies for controlling pertussis outbreaks.

Biologic and behavioral factors are known drivers of superspreading events of infectious diseases. In other words, the superspreading potential of an infector could be associated with the infectiousness of the infector and the number of contacts made by the infector (*29*). In this study, we identified that the *k* values were not significantly different between the persons vaccinated within 5 years and those vaccinated >5 years earlier. A previous study indicated that protection against pertussis wanes 5 years after the fifth dose of DTaP (*9*). Therefore, the number of contacts made by the case-patients could play a major role in increasing the risk for infection (i.e., increasing superspreading potential in this outbreak). However, because we were not able to quantify the exact number of contacts made per case, additional research with more detailed contact tracing data are warranted on how the number of contacts affects the superspreading events.

Waning immunity after acellular pertussis vaccination in children is known as a contributing factor to community pertussis transmission (*9*,*30*). Previous studies demonstrated a decline in immunity against pertussis beginning in the fourth year postvaccination (*10*); the protection against pertussis waned after the fifth dose of DTaP (*9*). However, the duration of protection after the sixth dose (i.e., Tdap vaccination), which was recommended between 11–12 years of age in South Korea, remains unclear (*2*). In this study, we could not identify a statistically significant trend of the estimated OR of acquiring pertussis infection per additional year after the sixth dose of vaccination. The likely reason for that finding is the limited sample size and variability in waning immunity and human behavior for the infection (i.e., biologic and behavioral factors) by persons over time. The age group most affected by pertussis in South Korea in 2024 was 13–14 years of age, with 25% overall incidence of pertussis, followed by 15–16 years (19% incidence), and 17–18 years (9%) (*31*); the highest vaccination coverage rate (83%) was in the 11–12 years age group. Study of the waning effectiveness of protection after the sixth dose of vaccination in a large sample size, with a detailed matrix of contact between infectors and infectees, would clarify our interpretations.

The overall attack rate in this study (10.1%) was larger than that of the previous outbreak (0.8%) in a South Korea school setting in 2017 (*32*). The increased attack rate in our study is likely a result of the different school settings (i.e., more physical activity in a sports boarding school). Furthermore, decreased immunity acquired from the reduced probability of being exposed to natural infection of pertussis in previous years (i.e., period of public health social measures implemented during the COVID-19 pandemic) is likely to affect the differences. Therefore, the recent increase in pertussis cases in many countries including South Korea may appear so because of fewer opportunities to become infected during the COVID-19 pandemic.

In 2023, South Korea immunization guidelines recommended either a Td or Tdap booster vaccination every 10 years for adults. However, this recommendation has not been fully incorporated into the National Immunization Program (*7*). Additional nationwide surveillance to support seroprevalence studies across different age groups could determine the exact burden of pertussis in South Korea and an effective booster administration schedule (*2*). Furthermore, developing new pertussis-containing vaccines that provide long-lasting immunity would reduce the burden of pertussis (*2*,*7*,*9*).

Our study estimated the serial interval and superspreading potential among persons who received a sixth dose of the pertussis vaccine in South Korea. One limitation of our work is that, although PCR is a sensitive and widely used diagnostic method for identifying *B. pertussis* infection, it can lead to false positives from other co-circulating respiratory pathogens (*33*). False positives could overestimate our results, including the attack rate and superspreading potential. Second, we based our serial interval estimates on self-reported data, which are not free from reporting (i.e., recall) bias, which could lead to either underestimation or overestimation of serial interval depending on the accuracy of symptom onset reporting. Third, natural immunity, acquired from previous *B. pertussis* infection, could introduce bias into our results (*2*). Persons with previous infection, in combination with vaccination, may exhibit different immunity profiles and transmission potential than those who only had vaccination without prior infection (*34*). The combination could affect our result of superspreading potential estimates and the vaccine effectiveness. Fourth, some cases may have been incorrectly attributed to clusters when the true infection source was elsewhere, which could affect our serial interval estimates and offspring distribution analysis. Fifth, our mean confirmation delay (i.e., the period between symptom onset and case confirmation) was 8 days; the delay with rapid isolation of suspected cases could shorten the serial interval by truncating the infectious period. Thus, the serial interval in our result may be underestimated. Sixth, we did not account for potential confounding factors, such as underlying conditions, which could modify the viral shedding dynamics and affect our results for attack rate and superspreading potential. Last, we could not examine the household secondary attack rate because we could not obtain the information for household composition of the case-patients and their household contacts. Further study is warranted to gain a more comprehensive understanding of pertussis transmission dynamics.

In conclusion, our study reveals the superspreading potential of pertussis among vaccinated children. Strict adherence to personal preventive measures, rapid case tracing, and isolation are essential to reduce community transmission of pertussis.

Appendix Additional information about transmission dynamics and parameters for pertussis during a school-based outbreak, South Korea, 2024.
