# Supplementary material for: Transmission Dynamics and Parameters for Pertussis during School-Based Outbreak, South Korea, 2024
Source: Emerg Infect Dis. 2025 Jul;31(7):1330–6. doi: 10.3201/eid3107.241643 (PMC12205447; doi:10.3201/eid3107.241643)
Supplement: Appendix — Additional information about transmission dynamics and parameters for pertussis during a school-based outbreak, South Korea, 2024. [file 24-1643-Techapp-s1.pdf]

Article DOI: https://doi.org/10.3201/eid3107.241643

EID cannot ensure accessibility for supplementary materials supplied by authors. Readers who have difficulty accessing supplementary content should contact the authors for assistance.

Transmission Dynamics and Parameters for Pertussis during School-Based Outbreak, South Korea, 2024

Appendix

Appendix Table 1. The dates of symptom onset among 48 cases. We retrieved the line-list data using an electronic data-extraction form (MS Excel software). This Excel spreadsheet column presents the exposure date, illness onset date, infection source, and demographic characteristics, including age and gender.

Source ID	Symptom onset	Sex	Age	Source of infection ID
1	2024–04–02	M	18	7
2	2024–04–06	M	13	19
3	2024–04–04	M	15	1
4	2024–04–15	M	15	1
5	2024–04–15	M	15	1
6	2024–04–10	M	17	1
7	2024–03–28	M	15	-
8	2024–04–04	M	15	1
9	2024–04–01	F	13	19
10	2024–04–10	M	12	2
11	2024–04–13	M	17	1
12	2024–04–16	F	18	1
13	2024–04–15	M	12	10
14	2024–04–16	M	12	18
15	2024–04–03	M	15	19
16	2024–04–13	M	16	19
17	2024–04–01	M	16	19
18	2024–04–10	M	13	19
19	2024–03–27	M	16	-
20	-	M	14	19
21	2024–04–16	M	13	14
22	2024–04–11	F	17	1
23	-	F	14	20
24	2024–04–16	M	12	19
25	2024–04–11	M	17	17
26	2024–04–03	M	17	19
27	-	F	17	12
28	2024–04–11	F	13	18
29	-	M	16	19
30	2024–04–17	M	17	1
31	2024–04–16	F	12	2
32	-	F	13	10
33	2024–04–19	F	13	18
34	2024–04–16	F	16	2
35	2024–04–15	M	16	2
36	2024–04–18	F	12	2
37	2024–04–21	M	15	2
38	2024–04–15	F	12	10
39	2024–04–19	F	16	7
40	2024–04–18	M	15	14
41	2024–05–03	F	17	15
42	2024–05–10	M	13	-
43	2024–05–17	M	13	10
44	2024–05–19	M	15	-

Source ID	Symptom onset	Sex	Age	Source of infection ID
45	2024–05–03	M	16	40
46	2024–05–23	M	16	44
47	2024–05–26	M	13	10
48	2024–05–28	M	17	43

Appendix Table 2. Estimation of serial interval distributions evaluated by fitting 3 different parametric distributions*

Characteristic	Log-normal	gamma	Weibull
Mean (Days)	10.01	9.47	9.45
Standard deviation	8.57	6.22	1.61
AIC	234.94	228.51	226.16

*AIC, Akaike information criterion.

Appendix Table 3. The offspring distribution estimations were evaluated by fitting a negative binomial distribution*

Characteristic	Negative binomial
Estimated Parameter	
R_t with 95% CI*	0.93 (95% CI: 0.41–1.85)
k with 95% CI*	0.23 (95% CI: 0.11–0.53)
Cases seeded 80% of transmissions (95% CI)	15.4% (95% CI: 8.0%–22.8%)
Log-likelihood	−55.73
AIC	115.46
BIC	119.12

*AIC, Akaike information criterion; BIC, Bayesian information criterion; CI, confidence interval.

Appendix Table 4. The offspring distribution estimations of two different times after the vaccination on infectors were evaluated by fitting a negative binomial distribution*

Characteristic	Negative binomial
Within 5 y	
Estimated parameter	
R_t with 95% CI	0.71 (95% CI: 0.29–1.40)
k with 95% CI	0.44 (95% CI: 0.12–10.27)
Log-likelihood	−32.40
AIC	68.80
BIC	71.46
Over 5 y	
Estimated parameter	
R_t with 95% CI	0.81 (95% CI: 0.31–1.70)
K with 95% CI	0.38 (95% CI: 0.11–6.00)
Log-likelihood	−32.14
AIC	68.28
BIC	70.79

*AIC, Akaike information criterion; BIC, Bayesian information criterion; CI, confidence interval.

Appendix Table 5. Waning of sixth dose of Tdap vaccination against acquiring pertussis*

	Ordered categorical variable model		Unordered categorical variable model	
Variable	Odds ratio with 95% CI	p value	Odds ratio with 95% CI	p value
Male	1.31 (0.68–2.65)	0.43	1.31 (0.68–2.65)	0.43
1st year	1.22 (0.08–9.38)	0.86	1.15 (0.64–4.11)	0.82
2nd year	0.90 (0.07–6.29)	0.92	0.36 (0.06–1.65)	0.19
3rd year	1.05 (0.09–8.01)	0.96	0.41 (0.10–1.57)	0.19
4th year	4.72 (0.42–48.46)	0.18	0.57 (0.18–1.95)	0.36
5th year	0.82 (0.10–7.84)	0.85	0.66 (0.22–2.16)	0.48
6th year	0.39 (0.05–4.28)	0.37	0.93 (0.19–4.03)	0.93
7th year	1.70 (0.19–19.48)	0.60	3.98 (0.28–57.42)	0.28
8th year	1.13 (0.13–10.77)	0.90	1.41 (0.28–6.43)	0.66
9th year	1.14 (0.10–8.20)	0.89	0.57 (0.05–3.34)	0.55
10th year	0.67 (0.07–5.54)	0.70	0.95 (0.09–6.17)	0.96
11th year	1.99 (0.26–22.06)	0.48	0.51 (0.05–3.34)	0.47
12th year	1.75 (0.32–15.21)	0.52	0.44 (0.00–5.31)	0.57
13th year	0.61 (0.13–3.42)	0.55	0.80 (0.01–11.96)	0.89
14th year	0.56 (0.13–2.48)	0.44	0.80 (0.01–11.96)	0.89
15th year	1.27 (0.23–6.94)	0.78	1.33 (0.01–28.94)	0.87

*To assess the waning immunity of the sixth dose of Tdap vaccination against pertussis infection, we estimated the odds ratio per time since the sixth dose of vaccination using two different models. In the ordered categorical model, time since the vaccination was categorized into ordinal levels assuming a structured trend in waning immunity across years post-vaccination. In the unordered categorical model, time since the vaccination was modeled as an unordered categorical variable to examine variation in pertussis risk over time without assuming a monotonic relationship. CI, Confidence Intervals.

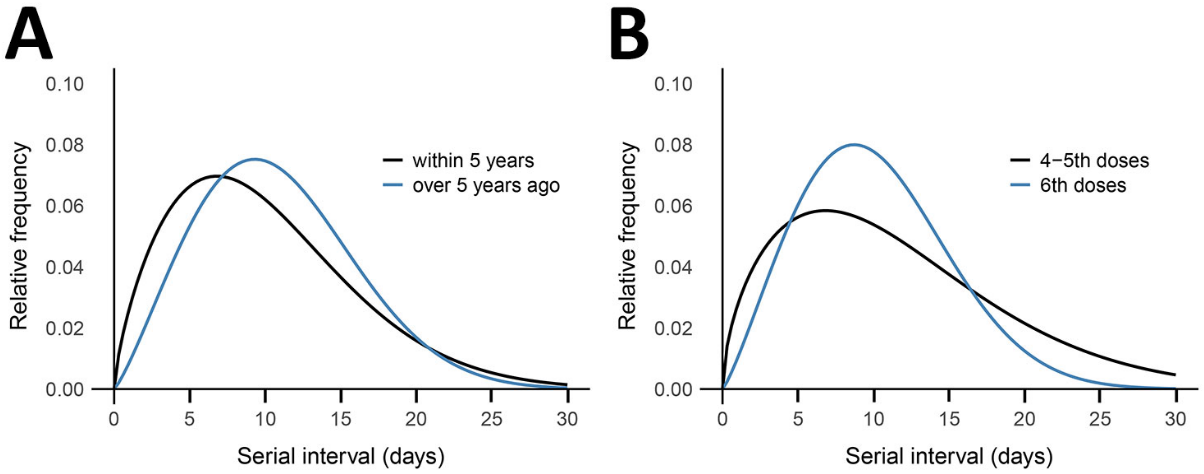

Appendix Figure. The difference in the serial interval by time since vaccination and by the infector's number of vaccination doses for pertussis. A) The solid black and blue lines represent the serial interval for infectors vaccinated within 5 years (n = 17) and those vaccinated over 5 years ago (n = 19), respectively. B) The solid black and blue lines represent the serial interval for infectors based on their final vaccination doses, with 7 individuals receiving doses 4–5 and 29 individuals receiving the 6th dose, respectively.
